# Impact of Preoperative Right Ventricular Function on Acute Renal Failure Development in Patients With Ventricular Assist Devices

**DOI:** 10.7759/cureus.72426

**Published:** 2024-10-26

**Authors:** Deniz S Beyazpınar, Mehmet Emir Erol

**Affiliations:** 1 Department of Cardiovascular Surgery, Baskent University Faculty of Medicine, Ankara, TUR; 2 Department of Cardiovascular Surgery, Hitit University School of Medicine, Çorum, TUR

**Keywords:** acute kidney injury, cardiac surgery procedures, renal insufficiency, right ventricular dysfunction. cardiac failure, ventricular assist device, ventricular dysfunction

## Abstract

Background

Acute kidney injury (AKI) and acute renal failure contribute significantly to the high mortality and morbidity seen in patients with left ventricular assist devices (LVADs). The role of preoperative right ventricular function in the onset of acute renal failure remains an area of active investigation. This study aims to explore the correlation between preoperative right ventricular function and the development of AKI in patients undergoing LVAD implantation.

Methods

The demographic characteristics, comorbidities, and preoperative and postoperative data of 61 patients who underwent LVAD implantation between April 2012 and December 2021 were carefully documented. Transthoracic echocardiography was used to assess right ventricular function, with tricuspid annular plane systolic excursion (TAPSE) and right ventricular fractional area change (FAC) serving as key evaluation parameters. TAPSE values of ≥17 mm and FAC of ≥35% were considered indicative of normal right ventricular function. Based on these measurements, the study analyzed the association between preoperative right ventricular function and the incidence of postoperative AKI.

Results

When patients were grouped based on TAPSE values, significant differences emerged in ICU stay duration, hospital stay length, AKI development, and the need for renal replacement therapy post-AKI. Elevated preoperative serum creatinine levels (p = 0.01), older age (p = 0.02), higher body surface area (p = 0.024), and lower TAPSE values (p = 0.02) were identified as risk factors for postoperative AKI. However, no significant correlation was found between preoperative FAC values and AKI incidence in LVAD-implanted patients.

Conclusions

Preoperative TAPSE appears to be a more reliable predictor of postoperative renal dysfunction than FAC.

## Introduction

Heart failure is a progressive condition that, if left untreated, carries a high mortality risk [1]. It affects around 1-2% of the population in developed countries, impacting approximately 23 million individuals globally [1,2]. While early stages of the disease can be managed with medical therapy, heart transplantation is recommended for patients with advanced heart failure who meet specific criteria [1]. Unfortunately, limited donor availability and a growing patient waiting list pose significant challenges.

To address these issues, the use of ventricular assist devices (VADs) is on the rise. Originally used as a bridge to transplantation (BTT), VADs are now also implemented for destination therapy, bridge to decision, and bridge to recovery [2,3]. Although BTT remains the primary use of VADs in Europe, this trend may shift soon [4]. In Japan, where heart transplant donors are particularly scarce, VADs have become the gold standard for managing end-stage heart failure [5]. However, prolonged use of VADs as destination therapy heightens the likelihood of complications.

End-stage heart failure often leads to other organ failures and dysfunctions. Acute kidney injury (AKI), for instance, occurs in 22-76% of patients with heart failure [6], significantly increasing mortality in both preoperative and postoperative periods. In nearly 50% of heart failure patients, the glomerular filtration rate is below 60 mL/min/1.73 m² [7,8]. Key hemodynamic factors contributing to renal impairment in advanced heart failure include reduced left ventricular output and elevated central venous pressure. Notably, studies identify renal dysfunction as a predisposing factor for right heart failure [9,10]. Right ventricular failure, which may develop after left VAD (LVAD) implantation, has been recognized as a potential complication [11].

In advanced heart failure, inadequate organ perfusion and high venous congestion are predominant issues. By sustaining arterial blood pressure postoperatively, LVADs enhance tissue and organ perfusion, helping to preserve organ functions. When right ventricular function is compromised, however, renal venous pressure increases, leading to elevated intratubular pressure, a reduction in net glomerular filtration pressure, and a subsequent decline in glomerular filtration rate [3,4]. Therefore, right ventricular function is a critical factor influencing prognosis, mortality, and morbidity in LVAD patients [11,12]. This study compares the incidence of AKI based on Kidney Disease: Improving Global Outcome (KDIGO) criteria between patients with normal and impaired preoperative right heart function [12].

## Materials and methods

From April 2012 to December 2021, 93 patients underwent LVAD implantation at our clinic. The devices used included HeartWare (HeartWare International Inc., Framingham, Massachusetts, USA) in 85 patients and Heartmate II and Heartmate III (Abbott Laboratories, Chicago, Illinois, USA) in four patients each. After obtaining ethics committee approval, the study was conducted in accordance with the Declaration of Helsinki. Following the application of exclusion criteria, 61 patients were enrolled in the study.

Exclusion criteria

Patients were excluded from the study if they met any of the following criteria: death within the first month post-surgery, heart transplant within one year of LVAD implantation, requirement of regular renal replacement therapy (RRT) due to chronic renal failure, need for extracorporeal membrane oxygenation or intra-aortic balloon pump support in the postoperative period, LVAD implantation due to congenital heart disease, or use of right ventricular or biventricular assist devices.

Our study has several limitations. Primarily, it is retrospective in design. Additionally, the lack of randomization across groups presents a significant limitation, as the selection of cardioplegia was surgeon-determined, introducing potential bias in patient selection (n = 32).

Study parameters

The following variables were retrospectively analyzed and recorded in the database: gender, age, body surface area (BSA), and the presence of comorbidities such as diabetes mellitus (DM), atrial fibrillation (AF), chronic obstructive pulmonary disease (COPD), and peripheral vascular disease. Additionally, the duration of intensive care and hospital stays, preoperative tricuspid annular plane systolic excursion (TAPSE) and right ventricular fractional area change (FAC) values, preoperative mean pulmonary artery pressure (PAP), pulmonary capillary wedge pressure (PCWP), and pulmonary vascular resistance (PVR) were documented. The development of AKI based on KDIGO criteria, its stage, the requirement for RRT, cardiopulmonary bypass times, and first-year mortality rates were also included in the analysis.

Determination of right ventricular dysfunction

The adequacy of right ventricular function is a critical consideration in patients with LVADs. Various techniques can be employed to assess right ventricular function, including echocardiography, cardiovascular magnetic resonance imaging, radionuclide imaging, computed tomography, and cardiac catheterization, all of which can aid in diagnosing right heart failure [13-15]. In this study, TAPSE and right ventricular FAC were utilized to evaluate right ventricular function. TAPSE values of ≥17 and FAC values of ≥35 were regarded as normal [14-16]. Based on these measurements, patients were categorized into two groups: those with low TAPSE values and those with normal TAPSE values, specifically forming a patient group with low FAC (LFAC) values and a patient group with normal FAC (NFAC) values.

Statistical analysis

Statistical analyses were performed using IBM SPSS Statistics for Windows, Version 22.0 (Released 2013; IBM Corp., Armonk, NY, USA). Intergroup comparisons were conducted using the chi-square test, Wilcoxon test, ANOVA, and Mann-Whitney U test, depending on their applicability. A p-value of <0.05 was considered statistically significant. Continuous variables were expressed as mean values with standard deviation, while categorical variables were presented as percentages.

Ethical statement

This study was approved by the Baskent University Institutional Review Board (approval number KA20/371) and received support from the Baskent University Research Fund. All protocols adhered to the ethical guidelines established in the 1975 Declaration of Helsinki.

## Results

Sixty-one patients who met the inclusion criteria were enrolled in the study, with 41 patients (67.2%) in the normal TAPSE (NTAPSE) group and 20 patients (32.8%) in the low TAPSE (LTAPSE) group. Additionally, based on FAC values, 49 patients (80.3%) were classified into the LFAC group, while 12 patients (19.7%) comprised the NFAC group.

The mean age of the cohort was 46.45 ± 4.95 years, with 55 male patients (90.16%). The mean BSA was 1.82 ± 0.38 m². DM and AF were present in 22 patients and 11 patients (18.03%), respectively. The mean serum creatinine (sCr), PAP, PCWP, and PVR levels were 1.15 ± 0.59 mg/dL, 35.91 ± 5.59 mmHg, 26.06 ± 2.93 mmHg, and 3.22 ± 1.35 Wood units, respectively. The demographic and preoperative parameters of patients in the TAPSE group are presented in Table 1. None of the demographic and preoperative parameters exhibited statistically significant differences between the LTAPSE and NTAPSE groups.

**Table 1:** Demographic and preoperative parameters of patients in the TAPSE group AF, atrial fibrillation; BSA, body surface area; DM, diabetes mellitus; PAP, pulmonary artery pressure; PCWP, pulmonary capillary wedge pressure; PVR, pulmonary vascular resistance; sCr, serum creatinine

Parameter	LTAPSE (n = 41)	NTAPSE (n = 20)	p-value
Age (year)	46.17 ± 4.67	47.1 ± 3.2	>0.05
Gender (male)	37 (90.25%)	18 (90%)	>0.05
BSA (m²)	1.75 ± 0.17	1.84 ± 0.31	>0.05
DM	14 (34.1%)	8 (40%)	>0.05
AF	7 (17.1%)	4 (20%)	>0.05
Preoperative sCr (mg/dL)	1.17 ± 0.49	1.11 ± 0.67	>0.05
Mean PAP (mmHg)	36.75 ± 4.74	34.84 ± 8.7	>0.05
PCWP (mmHg)	26.48 ± 2.51	25.2 ± 1.3	>0.05
PVR (Wood unit)	3.32 ± 1.51	3.03 ± 1.23	>0.05

The demographic and preoperative parameters of patients in the FAC group are presented in Table 2. Notably, a significant difference was observed in gender when comparing the LFAC and NFAC groups.

**Table 2:** Demographic and preoperative parameters of patients in the FAC group AF, atrial fibrillation; BSA, body surface area; DM, diabetes mellitus; PAP, pulmonary artery pressure; PCWP, pulmonary capillary wedge pressure; PVR, pulmonary vascular resistance; sCr, serum creatinine

Parameter	LFAC (n = 49)	NFAC (n = 12)	p-value
Age (year)	46.28 ± 4.78	47.08 ± 5.08	>0.05
Gender (male)	47 (95.9%)	8 (66.6%)	0.01
BSA (m²)	1.80 ± 0.12	1.87 ± 0.18	>0.05
DM	18 (36.7%)	4 (33.3%)	>0.05
AF	9 (21.9%)	2 (16.6%)	>0.05
Preoperative sCr (mg/dL)	1.11 ± 0.62	1.32 ± 0.42	>0.05
Mean PAP (mmHg)	35.64 ± 5.60	37.08 ± 5.22	>0.05
PCWP (mmHg)	25.46 ± 3.53	25.5 ± 2.12	>0.05
PVR (Wood unit)	3.03 ± 1.23	3.2 ± 1.76	>0.05

Postoperative parameters

The mean duration of cardiopulmonary bypass (CPB) for all patients was 133.57 ± 20.42 minutes. The mean lengths of stay in the intensive care unit and hospital were 22.78 ± 20.27 days and 35.37 ± 21.12 days, respectively. AKI developed in 25 patients (40.98%), with RRT required for 11 patients (18.03%). Additionally, nine patients (14.75%) died within the first year postoperatively. Perioperative and postoperative parameters for patients in the TAPSE group are presented in Table 3. Statistically significant differences were observed between the LTAPSE and NTAPSE groups concerning the length of stay in the intensive care unit, length of hospital stay, incidence of AKI, and necessity for RRT following AKI.

**Table 3:** Perioperative and postoperative parameters of patients in the TAPSE group AKI+, patients who developed acute kidney injury; CPB, cardiopulmonary bypass; LTAPSE, patient group with a TAPSE value below 17; NTAPSE, TAPSE value is 17 and higher patient group; RRT, renal replacement therapy

Parameter	LTAPSE (n = 41)	NTAPSE (n = 20)	p-value
Duration of CPB (min)	136.85 ± 17.14	126.85 ± 12.15	>0.05
Length of stay in intensive care unit (day)	27.73 ± 16.76	12.6 ± 20.4	0.03
Length of hospital stay (day)	41.26 ± 15.23	23.3 ± 23.7	0.01
AKI+	21 (51.22%)	4 (20%)	0.02
Need for RRT	11 (26.82%)	2 (10%)	0.04
One-year mortality	8 (19.51%)	1 (5%)	>0.05

The perioperative and postoperative parameters of patients in the FAC group are presented in Table 4. A comparison between the LFAC and NFAC groups revealed a statistically significant difference in both the length of hospital stay and the duration of stay in the intensive care unit.

**Table 4:** Perioperative and postoperative parameters of patients in the FAC group AKI+, patients who developed acute kidney injury; CPB, cardiopulmonary bypass; LFAC, patient group with a FAC value below 35; NFAC, FAC value is 35 and higher patient group; RRT, renal replacement therapy

Parameter	LFAC (n = 49)	NFAC (n = 12)	p-value
Duration of CPB min)	133.53 ± 20.46	133.75 ± 14.25	>0.05
Length of stay in intensive care unit (day)	24.1 ± 20.39	17.33 ± 24.16	0.04
Length of hospital stay (day)	38.08 ± 18.41	24.33 ± 24.16	0.02
AKI+	19 (38.8%)	6 (50%)	>0.05
Need for RRT	10 (20.41%)	3 (25%)	>0.05
One-year mortality	9 (18.36%)	0 (0%)	>0.05

The mean preoperative sCr levels for patients in the AKI+ group were 1.54 ± 0.32 mg/dL, while those in the non-AKI group had levels of 0.88 ± 0.45 mg/dL (p = 0.001). Preoperative elevation in sCr levels is identified as a risk factor for the development of AKI.

Furthermore, the preoperative mean sCr levels of patients who required RRT were 1.99 ± 0.83 mg/dL compared to 0.92 ± 0.37 mg/dL for those who did not require RRT (p = 0.01), indicating that elevated preoperative sCr levels are also a risk factor for the need for RRT.

Additional risk factors for AKI included high preoperative sCr levels (p = 0.01), age (p = 0.02), BSA (p = 0.024), and low TAPSE values (p = 0.02). Patients with AKI experienced longer lengths of stay in both the intensive care unit and the hospital (p = 0.01), with these extended durations being statistically significant.

## Discussion

The main finding of this study suggests that TAPSE values obtained through preoperative transthoracic echocardiography, which assess right ventricular function, may serve as a more effective marker than FAC measurements in predicting the risk of AKI and the necessity for RRT.

Numerous studies have shown that preoperative right ventricular function is associated with postoperative mortality and morbidity [9,17]. However, only a few studies have utilized TAPSE to evaluate preoperative right ventricular function. While Guven et al. [18] demonstrated an association between FAC values and postoperative AKI, our study did not find such a relationship. Parikh et al. [19] highlighted that the need for RRT increases in the postoperative period among patients with preoperative right ventricular failure. Our findings align with this, revealing a significantly higher incidence of postoperative AKI and the need for RRT in patients with low TAPSE values compared to those with normal TAPSE values.

We identified elevated preoperative sCr levels as a risk factor for the development of AKI and the subsequent need for RRT. In our study, 13 patients (12.3%) required RRT, a rate consistent with findings from previous studies [19,20].

Preoperative parameters and comorbidities, including gender, DM, AF, COPD, FAC values, PVR measurements, PCWP, PAP, and the duration of CPB. were not associated with the development of AKI or the requirement for RRT. Similarly, previous research has indicated that the presence of DM does not increase the risk of developing AKI [10,19]. Naeije and Manes [20] noted that pulmonary hypertension raises right ventricular afterload, potentially leading to future right heart failure; however, in our study, the preoperative mean PAP was not identified as a risk factor for AKI or RRT needs in the postoperative period.

Kirklin et al. [21] conducted a retrospective analysis using the INTERMACS database and found that patients with renal dysfunction prior to LVAD implantation exhibited higher mortality rates, longer hospitalization, and lower survival in the post-implantation period. Our study corroborates this finding, showing that lengths of hospital and intensive care stays were significantly longer for patients with AKI who required RRT post-AKI development. Based on these observations and those reported by Kirklin et al., we conclude that patients who develop AKI incur higher healthcare expenditures due to increased lengths of stay; however, there was no difference in one-year survival between patients who experienced AKI and required RRT in the early postoperative period and those who did not. Additionally, Kirklin et al. [21] identified preoperative renal dysfunction, high-dose diuretic use, the need for inotropic support, and the requirement for dialysis as risk factors for mortality. Notably, the need for preoperative dialysis increased mortality among these patients by 2.37 times [21].

Right ventricular failure in patients with LVADs is associated with increased postoperative mortality [21]. In our study, one patient in the normal TAPSE group and eight patients in the low TAPSE group died within the first postoperative year. Among those in the LFAC group, nine patients died, while no deaths were observed in the NFAC group. Although mortality was numerically higher in patients with low TAPSE and FAC values compared to those with normal values, the difference was not statistically significant. We attribute this observation to the small sample size of the patient group included in the study.

Limitations

This study has limitations. LVAD implantation is conducted in a limited number of centers in our country, resulting in a low patient sample size for this single-center study. Furthermore, the COVID-19 pandemic significantly impacted healthcare systems worldwide, particularly affecting patients with end-stage heart failure, as many hospitals suspended operations during that time. Consequently, multicenter studies with larger patient populations are needed in the future to obtain more comprehensive and beneficial results.

## Conclusions

Mortality and morbidity associated with right ventricular dysfunction following LVAD implantation is a significant concern. Patients undergoing LVAD implantation for destination therapy should ideally experience a high quality of life, making the selection of appropriate candidates crucial for ensuring their long-term survival. Our study demonstrates that preoperatively measured TAPSE values serve as a reliable parameter for predicting renal dysfunction that may arise in the postoperative period. Additionally, we identified advanced age, high BSA, and elevated preoperative sCr levels as risk factors for the development of AKI and the need for RRT in the postoperative period.
